# Supplementary material for: New Genetic Variants in CYP2B6 and SLC6A Support the Role of Oxidative Stress in Familial Ménière’s Disease
Source: Genes (Basel). 2022 Jun 1;13(6):998. doi: 10.3390/genes13060998 (PMC9222295; doi:10.3390/genes13060998)
Supplement: Supplementary file 1 [file genes-13-00998-s001.zip › genes-1723846-supplementary.pdf]

Table S1. Summary of the whole exome sequencing data. Whole exome sequencing resulted on average in 7 346 Mb raw bases from the Illumina sequencer. After removing low-quality reads we obtained averagely 73 383 061 clean reads (7 3719 Mb). The clean reads of each sample had high Q20 and Q30 . which showed high sequencing quality. The average GC content was 48.55%.

Family	Raw reads	Raw bases (Mb)	Clean reads	Clean bases (Mb)	Clead data rate (%)	Clead read1 Q20 (%)	Clead read2 Q20 (%)	Clead read1 Q30 (%)	Clead read2 Q30 (%)	GC content (%)
I/III:3	63 537 348	6353.73	63 236 416	6243.53	98.27	97.46	94.33	93.26	88.54	47.03
I/IV:1	76 017 748	7601.78	76 001 674	7597.91	99.95	98.59	96.54	95.67	91.84	49.22
I/II:3	82 634 223	8263.42	82 616 724	8259.08	99.95	98.62	96.25	95.76	91.34	48.44
I/II:2	64 023 450	6402.31	63 490 334	6208.32	96.97	96.93	93.74	92.17	87.74	47.96
II/I:2	71 304 740	7130.47	71 290 062	7126.17	99.94	98.64	96.62	95.73	91.91	50.47
II/II:2	77 449 710	7744.97	77 433 270	7740.88	99.95	98.70	97.11	95.86	92.90	51.00
II/II:1	67 099 556	6709.95	67 086 134	6706.76	99.95	98.62	95.74	95.73	90.25	49.20
II/III:1	77 130 836	7713.08	77 115 032	7707.99	99.93	98.69	97.17	95.81	92.95	51.65
III/I:2	79 105 149	7910.51	79 095 074	7907.36	99.96	98.67	96.93	95.89	92.72	47.38
III/II:1	75 291 247	7529.12	75 273 798	7525.49	99.95	98.65	96.82	95.85	92.58	47.33
III/I:1	81 292 537	8129.25	81 281 430	8126.34	99.96	98.62	96.11	95.81	91.23	46.19
III/II:2	75 415 717	7541.57	75 405 678	7538.79	99.96	98.60	96.32	95.76	91.65	45.92
III/II:3	64 672 394	6467.24	64 654 168	6464.15	99.95	98.59	94.99	95.57	88.98	49.36

Table S2. Statistics of alignment. Clean reads were aligned to the human reference genome hg19 using Burrows-Wheeler Aligner (BWA). On average. 99.29 % mapped succesfully. The mean sequencing depth on target regions were 71-fold. On average per sequencing individual. 97% of the targeted bases had at least 10x coverage.

Family/ID	Initial bases on target	Total effective reads	Total effective bases (Mb)	Effective sequences on target (Mb)	Capture spesificity (%)	Mapping rate in target region (%)	Mismatch rate in target region (%)	Average sequencing depth
I/III:3	51 189 318	56 307 966	5 351.32	3 040.67	56.82	96.84	0.39	59.4
I/IV:1	51 189 318	65 643 362	6 525.99	3 660.27	56.09	99.71	0.31	71.5
I/II:3	51 189 318	70 193 218	6 974.25	3 887.17	55.74	99.71	0.32	75.94
I/II:2	51 189 318	59 285 752	5 081.56	2 740.50	53.93	96.43	0.44	53.54
II/I:2	51 189 318	62 891 666	6 258.28	3 697.57	59.08	99.74	0.31	72.23
II/II:2	51 189 318	68 484 275	6 817.70	4 132.41	60.61	99.76	0.29	80.73
II/II:1	51 189 318	58 161 440	5 779.54	3 270.11	56.58	99.7	0.34	63.88
II/III:1	51 189 318	68 095 080	6 779.97	4 137.94	61.03	99.76	0.28	80.84
III/I:2	51 189 318	69 232 621	6 886.43	4 074.81	59.17	99.89	0.29	79.6
III/II:1	51 189 318	65 790 225	6 540.89	3 755.00	57.41	99.87	0.30	73.36
III/I:1	51 189 318	69 057 221	6 862.29	3 814.88	55.59	99.82	0.33	74.52
III/II:2	51 189 318	65 030 538	6 464.97	3 596.99	55.64	99.83	0.32	70.27
III/II:3	51 189 318	58 528 259	5 815.88	3 405.35	58.55	99.71	0.37	66.52

Table S3. Quality statistics for each identified variant.

Chr	Position	Ref	Alt	QC	Variant info		
**FAMILY I**							
chr19	41509934	C	T	PASS	AC=1;AF=0.500;AN=2;BaseQRankSum=2.17;ClippingRankSum=-2.096e+00;DP=21;FS=6.410;GQ_MEAN=275.00;MLEAC=1; MLEAF=0.500;MQ=52.71;MQ0=0;MQRankSum=-1.070e-01;NCC=0;QD=11.75;ReadPosRankSum=2.24;SOR=2.833	GT:AD:DP:GQ:PL	0/1:12.9:21:99:275.0.322
**FAMILY II**							
chr7	65445284	G	A	PASS	AC=1;AF=0.500;AN=2;BaseQRankSum=1.51;ClippingRankSum=-1.530e-01;DP=81;FS=15.822;GQ_MEAN=815.00;MLEAC=1;MLEAF=0.500; MQ=60.00;MQ0=0;MQRankSum=2.17;NCC=0;QD=19.17;ReadPosRankSum=0.320;SOR=0.046	GT:AD:DP:GQ:PL	0/1:29.52:81:99:1581.0.815
chr15	43500504	C	T	PASS	AC=1;AF=0.500;AN=2;BaseQRankSum=-5.710e-01;ClippingRankSum=-3.060e-01;DP=84;FS=9.570;GQ_MEAN=1042.00;MLEAC=1;MLEAF=0.500; MQ=60.00;MQ0=0;MQRankSum=1.04;NCC=0;QD=12.21;ReadPosRankSum=2.05;SOR=1.711	GT:AD:DP:GQ:PL	0/1:45.38:83:99:1042.0.1317
**FAMILY II**							
chr12	29630096	A	T	PASS	AC=1;AF=0.500;AN=2;BaseQRankSum=0.146;ClippingRankSum=-6.390e-01;DP=98;FS=1.728;GQ_MEAN=1221.00;MLEAC=1;MLEAF=0.500; MQ=60.00;MQ0=0;MQRankSum=-5.400e-02;NCC=0;QD=12.17;ReadPosRankSum=0.532;SOR=0.931	GT:AD:DP:GQ:PL	0/1:54.44:98:99:1221.0.1586
chr12	123089490	A	C	PASS	AC=1;AF=0.500;AN=2;BaseQRankSum=-4.086e+00;ClippingRankSum=0.349;DP=79;FS=4.963;GQ_MEAN=887.00;MLEAC=1;MLEAF=0.500; MQ=60.00;MQ0=0;MQRankSum=0.177;NCC=0;QD=18.12;ReadPosRankSum=0.510;SOR=0.274	GT:AD:DP:GQ:PL	0/1:30.49:79:99:1460.0.887
chr1	197059082	T	C	PASS	AC=1;AF=0.500;AN=2;BaseQRankSum=-8.080e-01;ClippingRankSum=-4.660e-01;DP=65;FS=0.974;GQ_MEAN=903.00;MLEAC=1;MLEAF=0.500; MQ=60.00;MQ0=0;MQRankSum=-1.438e+00;NCC=0;QD=14.57;ReadPosRankSum=-7.680e-01;SOR=0.495	GT:AD:DP:GQ:PL	0/1:31.34:65:99:975.0.903
chr5	149576383	G	C	PASS	AC=1;AF=0.500;AN=2;BaseQRankSum=-1.796e+00;ClippingRankSum=-9.510e-01;DP=31;FS=1.544;GQ_MEAN=308.00;MLEAC=1;MLEAF=0.500; MQ=60.00;MQ0=0;MQRankSum=-1.542e+00;NCC=0;QD=17.51;ReadPosRankSum=1.16;SOR=1.230	GT:AD:DP:GQ:PL	0/1:10.21:31:99:571.0.308

Table S4. Variant annotations.

Chr	Position	Ref	Alt	Locus	Gene	Expression in inner ear database	SISU (N)	SISU AF	Exonic Function	AAChange.refGene	1000g 2015aug eur	snp142	Kaviar AF	Kaviar AC	Kaviar AN	ExAC ALL	ExAC FIN	SIFT score	SIFT pred	Polyphen2 (PP2) HDIV Score	PP2 HDIV pred	PP2 HVAR score	PP2 HVAR pred
**FAMILY I**																							
chr19	41509934	C	T	exonic	CYP2B6	YES	4/10486	0.000001	nonsynonymous	CYP2B6:NM_000767: exon2:c.C200T:p.T67M	0.002	rs138264188	0.001216	188	154602	0.0013	0.0002	0.016	D	1	D	1	D
**FAMILY II**																							
chr7	65445284	G	A	exonic	GUSB	YES	3/8559	0.00035	nonsynonymous	GUSB:NM_000181:exon2:c.C323T:p.P108L.GUSB: NM_001284290:exon2:c.C323T:p.P108L	.	NA	NA	NA	NA	NA	NA	0.001	D	1	D	1	D
chr15	43500504	C	T	exonic	EPB42	YES	21/10457	0.002	stopgain	EPB42:NM_000119:exon8:c.G1089A:p.W363X. EPB42:NM_001114134:exon8:c.G999A:p.W333X	.	rs201351228	7.76E-05	12	154602	9.95E-05	0.0019	NA	NA	NA	NA	NA	NA
**FAMILY III**																							
chr12	29630096	A	T	exonic	OVCH1	NA	10/10468	0.00096	stopgain	OVCH1:NM_183378:exon12:c.T1316A:p.L439X	.	.	5.17E-05	8	154602	6.68E-05	0.0006	NA	NA	NA	NA	NA	NA
chr12	123089490	A	C	exonic	KNTC1	YES	4/10394	0.00038	nonsynonymous	KNTC1:NM_014708:exon50:c.A5242C:p.T1748P	.	NA	NA	NA	NA	NA	NA	0	D	1	D	1	D
chr1	197059082	T	C	exonic	ASPM	YES	NA	NA	nonsynonymou	ASPM:NM_001206846:exon24:c.A5207G:p.Q1736R ASPM:NM_018136:exon25:c.A9962G:p.Q3321R	.	NA	NA	NA	NA	NA	NA	0.315	T	1	D	0.96	D
chr5	149576383	G	C	exonic	SLC6A7	NA	27/10463	0.0026	nonsynonymous	SLC6A7:NM_014228:exon3:c.G322C:p.V108L	.	NA	5.17E-05	8	154602	6.61E-05	0.0011	0.024	D	0.843	P	0.737	P

LRT score	LRT pred	Mutation Taster score	Mutation Taster pred	Mutation Assessor score	Mutation Assessor pred	FATHMM score	FATHMM pred	PROVEAN score	PROVEAN pred	VEST3 score	CADD raw	CADD phred	DANN score	Fathmm MKL Codings core	Fathmm MKL Coding pred	Meta SVM score	Meta SV pred	MetaLR score	MetaLR pred	Integrated fitCons score	Integrated Confidence value	GERP++ RS	phyloP 7way vertebrate	phyloP 20way mammalian	SiPhy 29way logOdds	ljb2 pp2hdiv
**FAMILY I**																										
0	D	0.981	N	3.23	M	-0.48	T	-5.04	D	0.289	5.681	26.7	0.999	0.801	D	-0.279	T	0.553	D	0.487	0	4.15	0.824	0.807	7.857	1.0.D
**FAMILY II**																										
0	D	1	D	2.385	M	-4.04	D	-8.24	D	0.899	5.709	26.8	0.999	0.97	D	1.081	D	0.935	D	0.707	0	5.22	0.897	1.011	17.788	1.0.D
0	D	1	A	NA	NA	NA	NA	NA	NA	NA	12.266	39	0.994	0.959	D	NA	NA	NA	NA	0.497	0	5.77	0.871	0.935	17.836	NA
**FAMILY III**																										
NA	NA	1	A	NA	NA	NA	NA	NA	NA	NA	8.122	35	0.966	0.125	N	NA	NA	NA	NA	0.554	0	0.327	0.423	0.079	6.189	NA
0	D	1	D	2.175	M	0.55	T	-4.23	D	0.981	6.018	27.9	0.997	0.994	D	-0.327	T	0.343	T	0.732	0	5.71	1.062	1.14	15.989	1.0.D
0	D	0.996	N	2.015	M	0.89	T	-1.48	N	0.698	5.654	26.7	0.999	0.98	D	-0.87	T	0.185	T	0.732	0	5.86	0.991	1.061	11.352	1.0.D
0	D	1	D	2.005	M	-0.87	T	-2.27	N	0.851	4.244	23.9	0.998	0.987	D	0.058	D	0.513	D	0.497	0	5.32	0.917	1.048	18.992	0.843.P

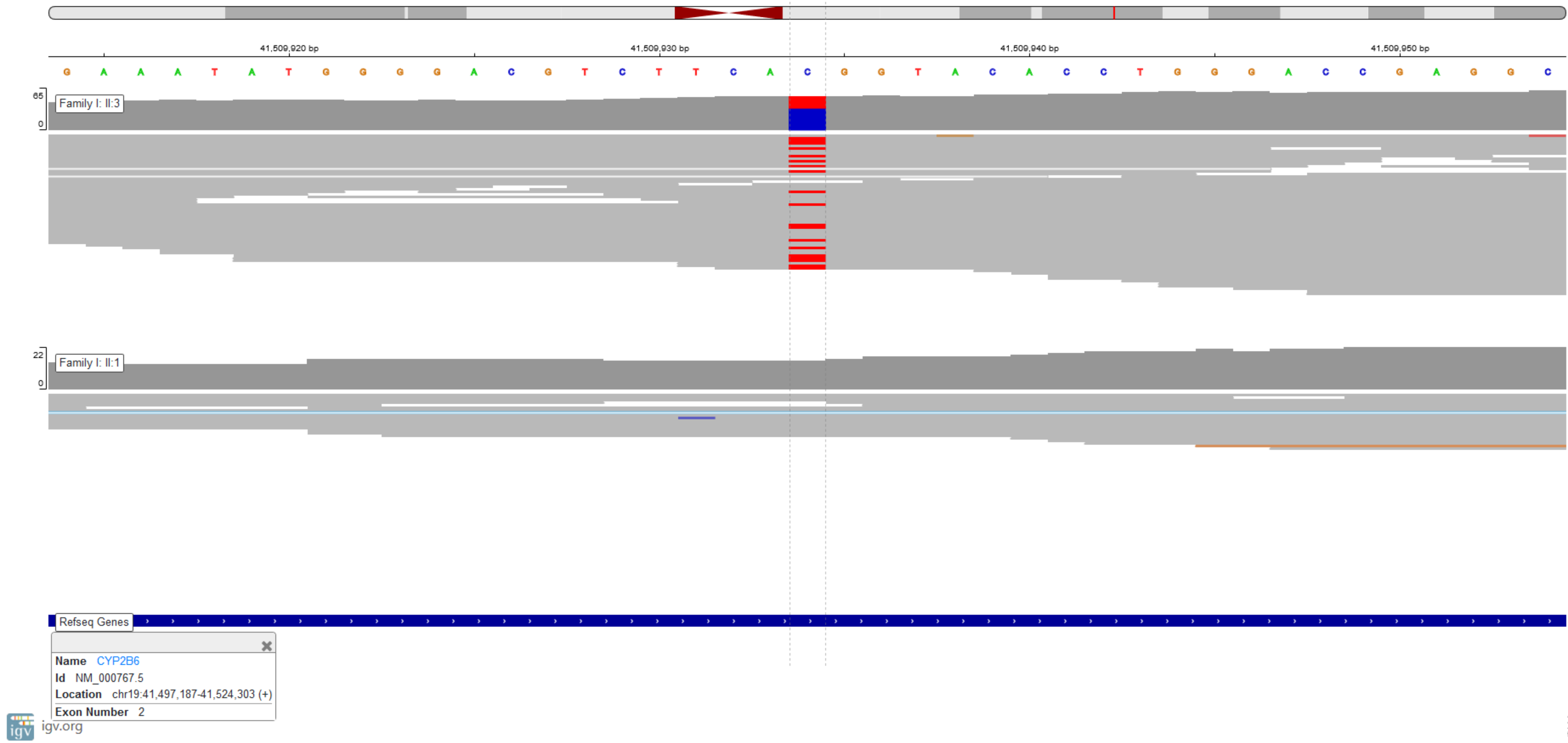

Figure S1. Visualization of the locus 19:41509934 (hg19) in the Family I . The variant c.200C>T is located in the *CYP2B6* gene (+ strand).
Locus is visualized form one MD case carrying the variant (II:3) and one healthy family member not carrying the variant (II:1). Interactive genomics viewer (IGV) was used to visualize the data.

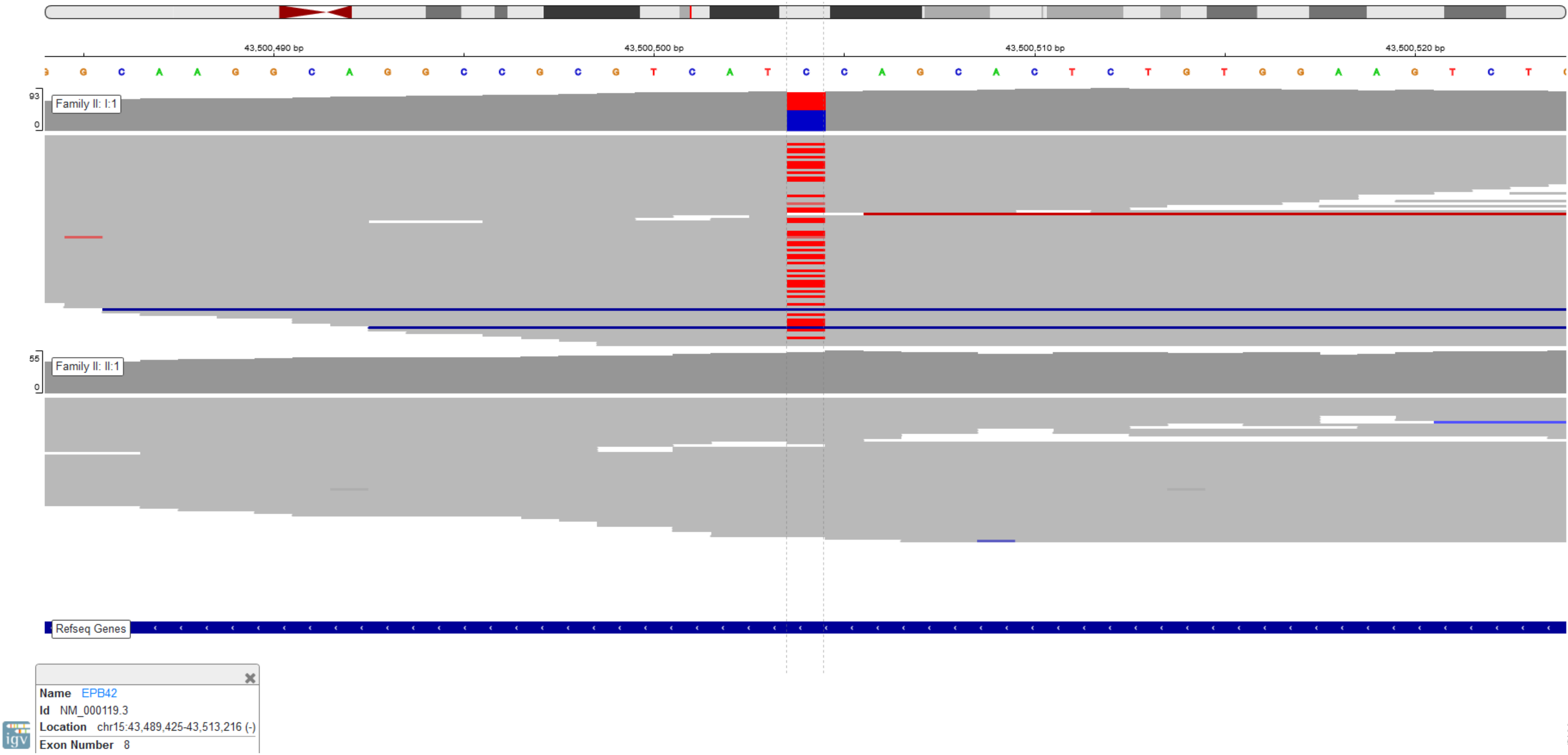

Figure S2. Visualization of the locus chr15:43500504 (hg19) in the Family II. The variant c.1089G>A is located in the *EPB42* gene (- strand).
Locus is visualized form one MD case carrying the variant (I:1) and one healthy family member not carrying the variant (II:1). Interactive genomics viewer (IGV) was used to visualize the data.

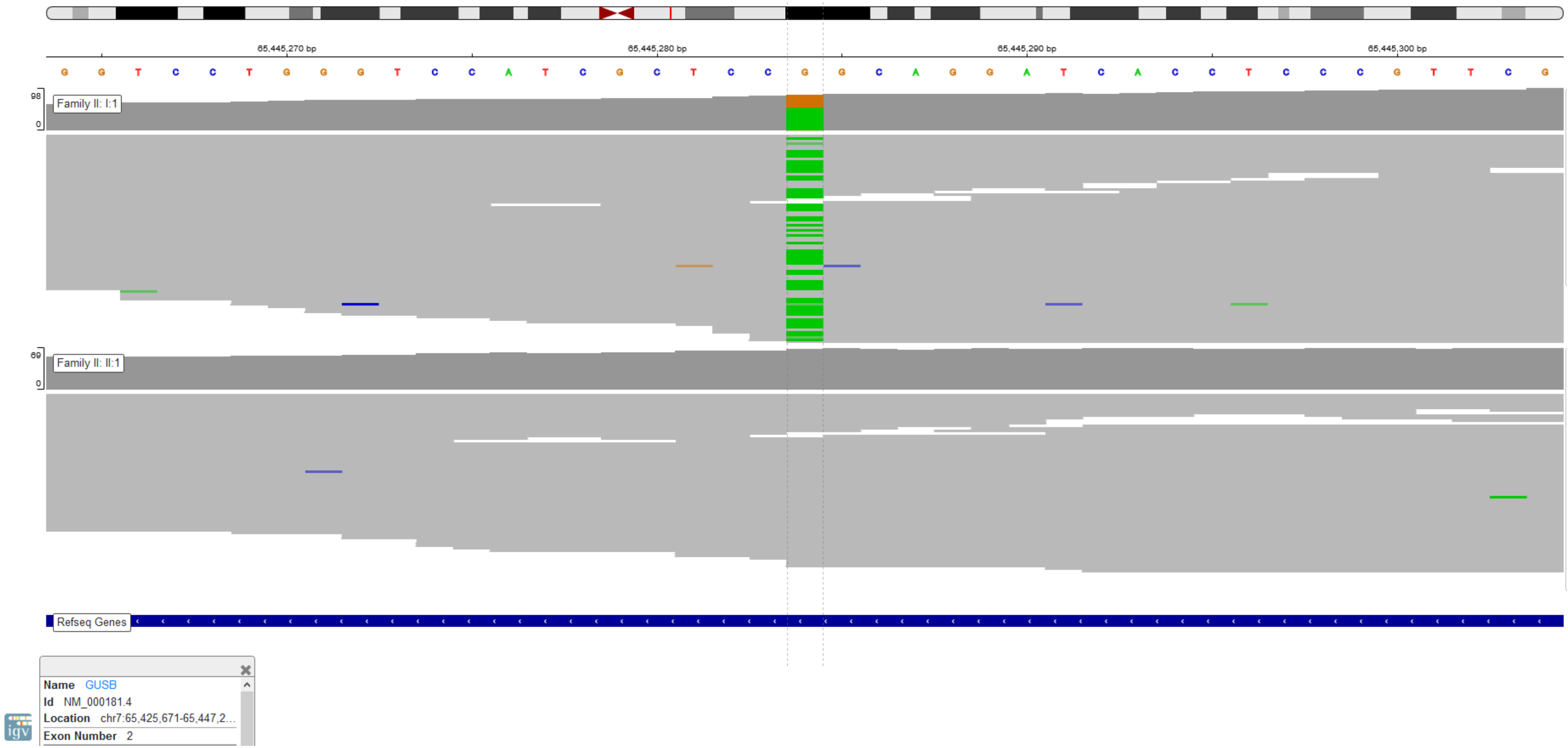

Figure S3. Visualization of the locus chr7:65445284 (hg19) in the Family II. The variant c.323C>T is located in the *GUSB* gene (- strand).
Locus is visualized form one MD case carrying the variant (I:1) and one healthy family member not carrying the variant (II:1). Interactive genomics viewer (IGV) was used to visualize the data.

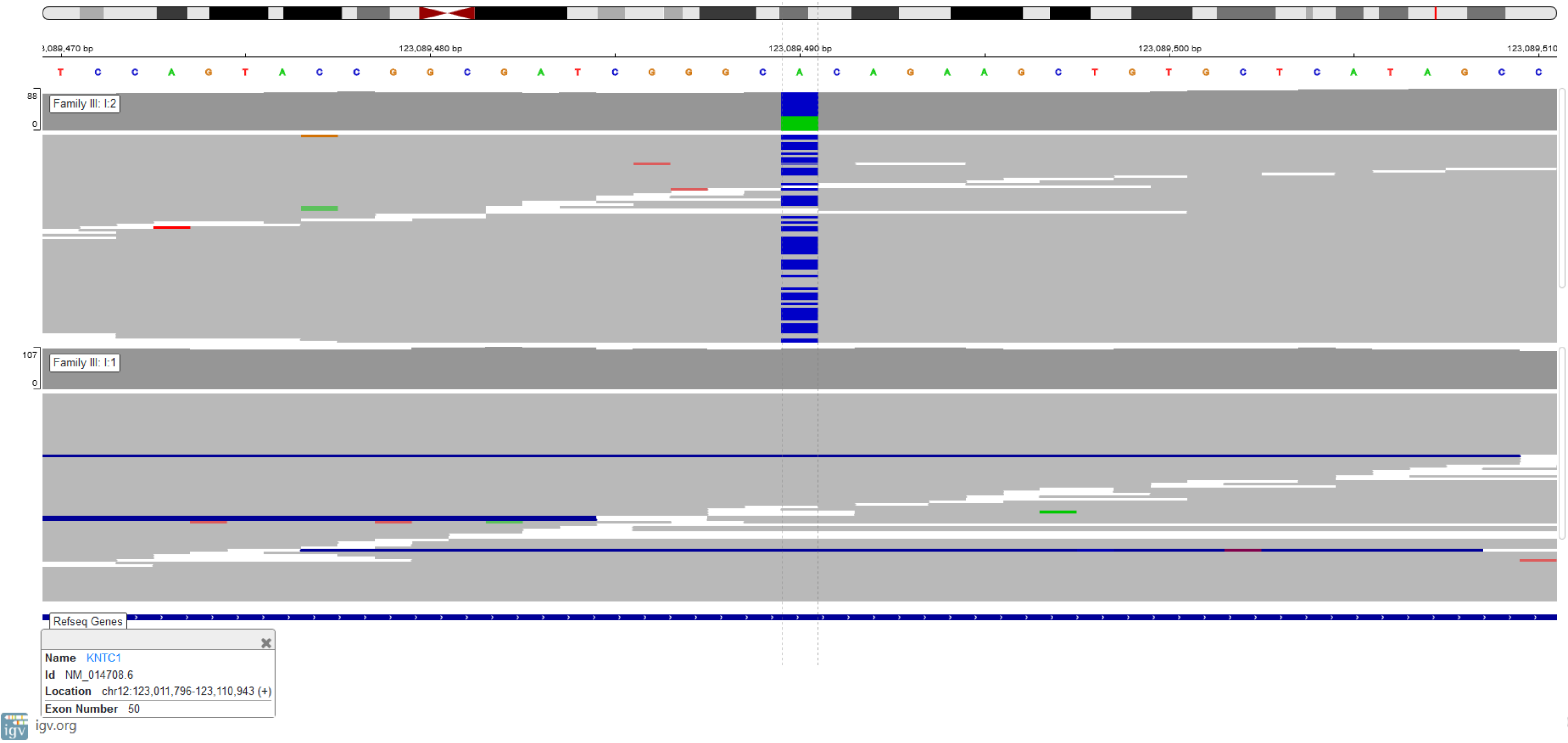

Figure S4. Visualization of the locus chr12:123089490 (hg19) in the Family III. The variant c.5242A>C is located in the *KNTC1* gene (+ strand).
Locus is visualized form one MD case carrying the variant (I:2) and one healthy family member not carrying the variant (I:1). Interactive genomics viewer (IGV) was used to visualize the data.

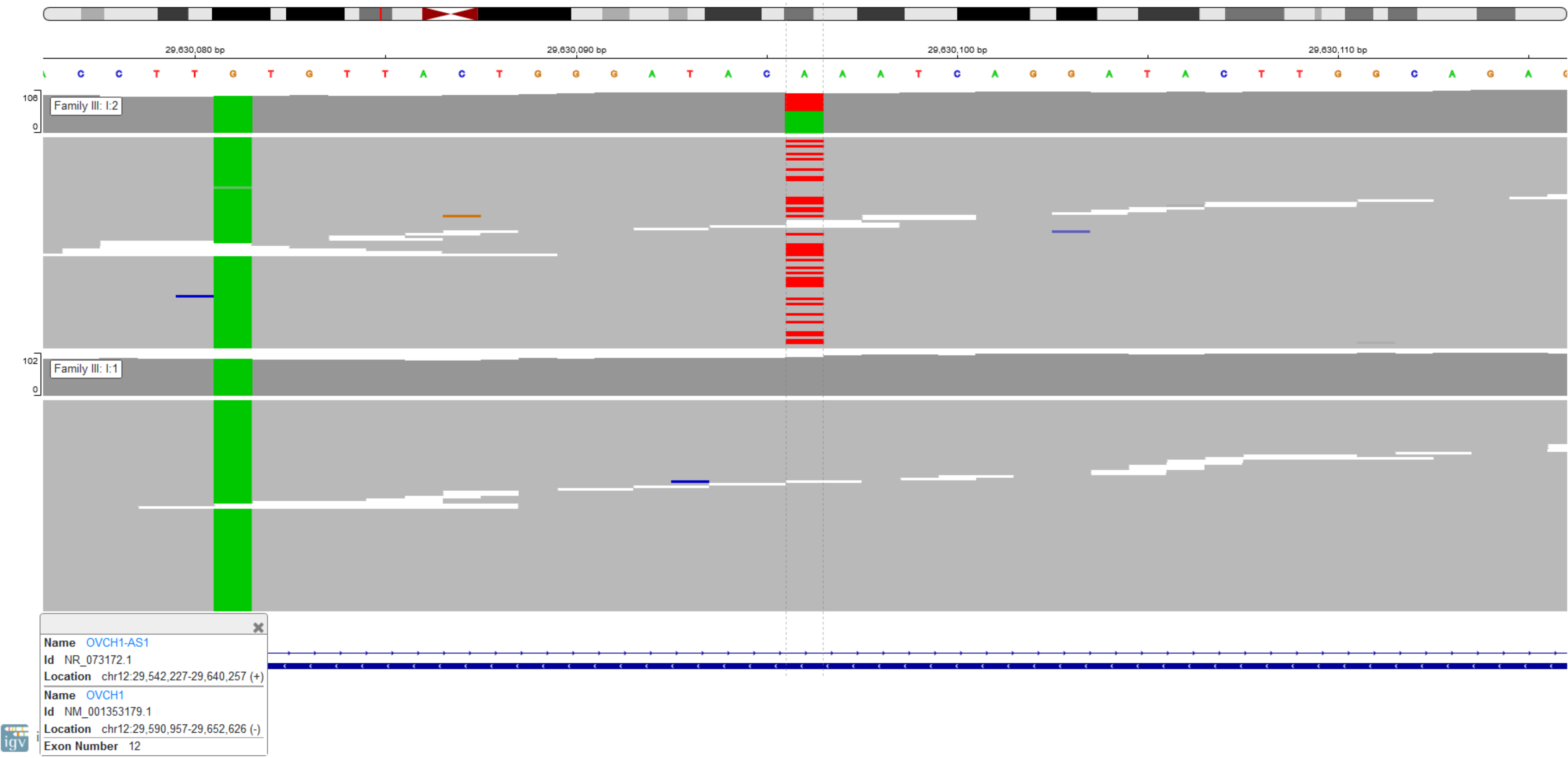

Figure S5. Visualization of the locus chr12:29630096 (hg19) in the Family III. The variant c.1316T>A is located in the *OVCH1* gene (+ strand).
Locus is visualized form one MD case carrying the variant (I:2) and one healthy family member not carrying the variant (I:1). Interactive genomics viewer (IGV) was used to visualize the data.

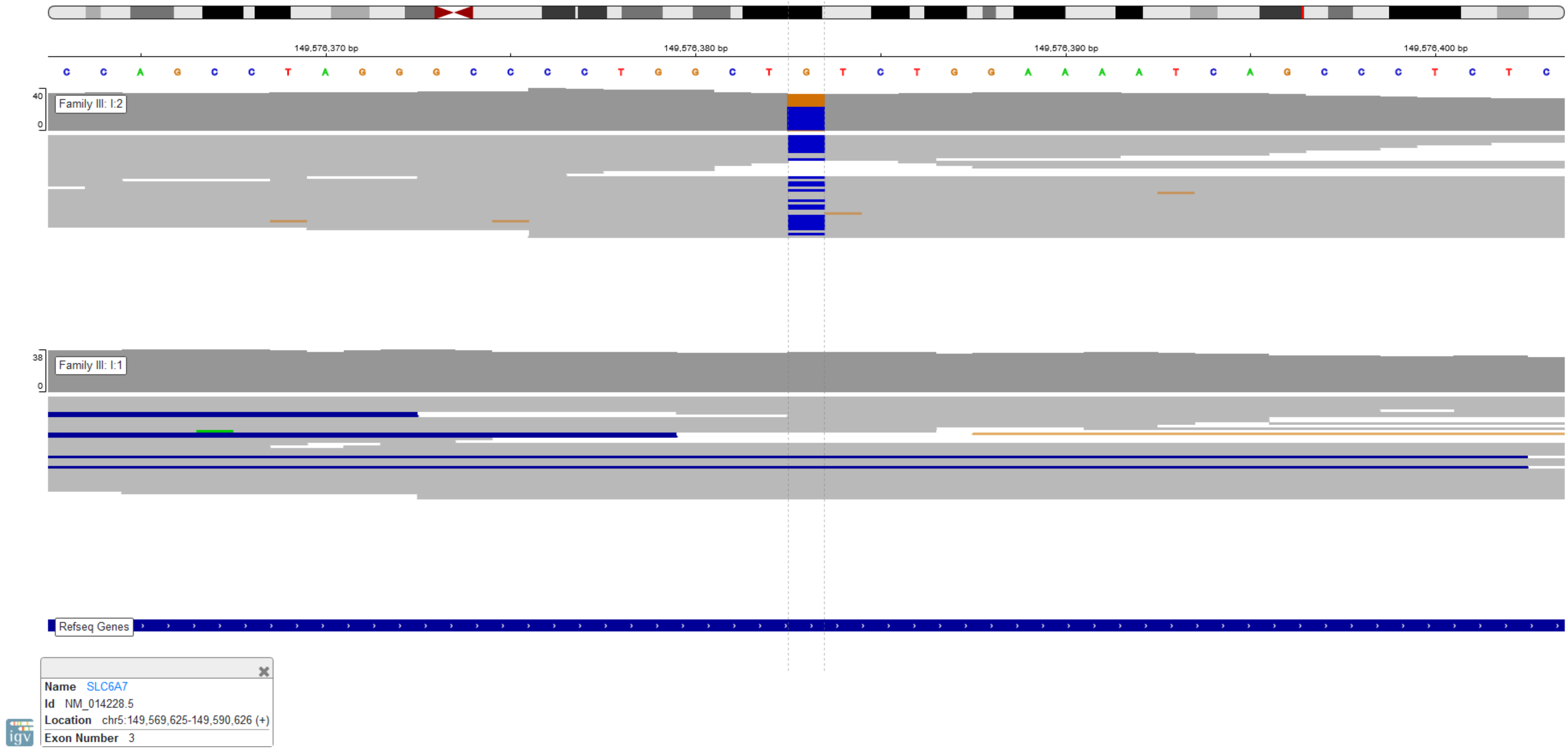

Figure S6. Visualization of the locus chr5:149576383 (hg19) in the Family III. The variant c.322G>C is located in the *SLC6A7* gene (+ strand).
Locus is visualized form one MD case carrying the variant (I:2) and one healthy family member not carrying the variant (I:1). Interactive genomics viewer (IGV) was used to visualize the data.

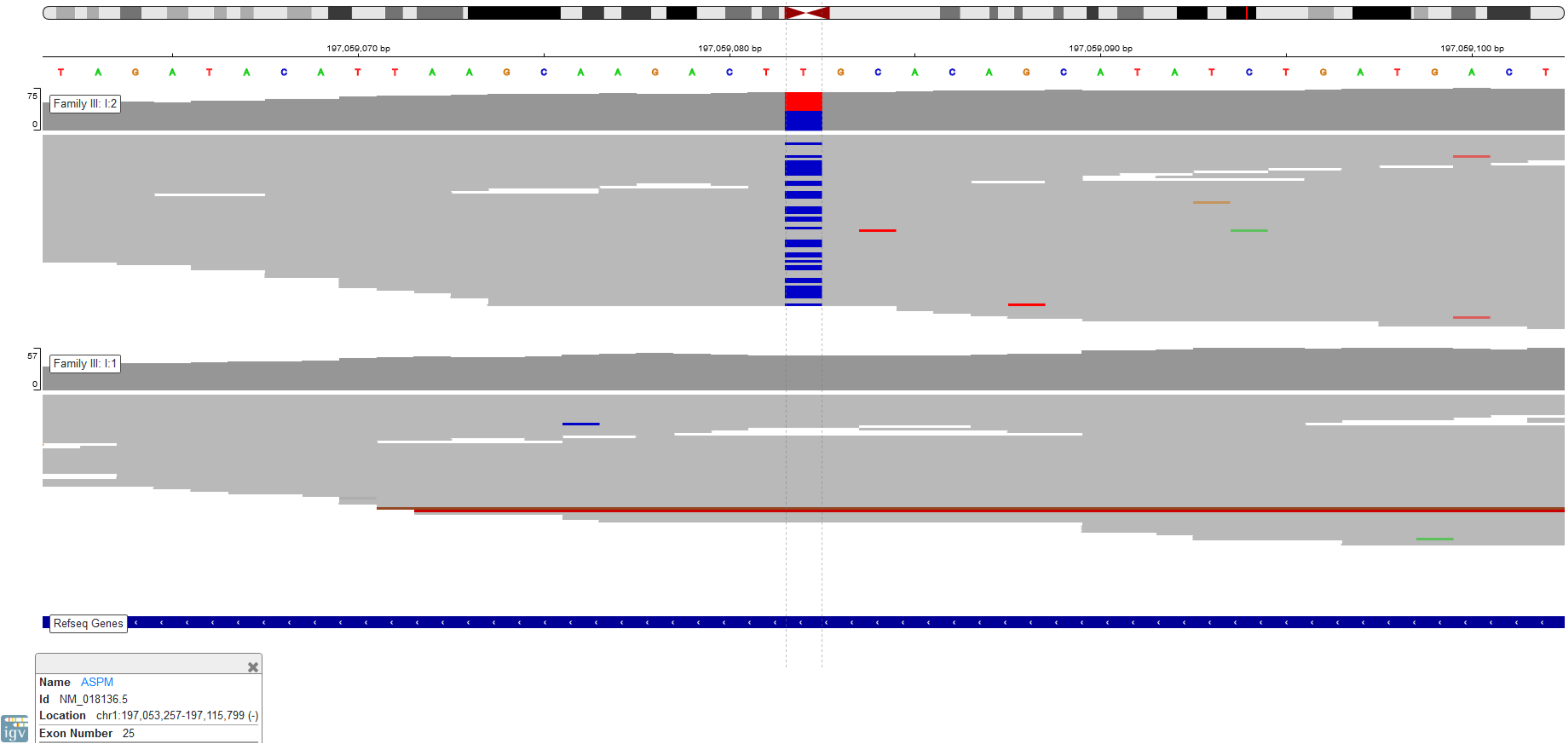

Figure S7. Visualization of the locus chr1:197059082 (hg19) in the Family III. The variant c.5207A>G is located in the *ASPM* gene (strand).
Locus is visualized form one MD case carrying the variant (I:2) and one healthy family member not carrying the variant (I:1). Interactive genomics viewer (IGV) was used to visualize the data.
